# UBE2C promotes myoblast differentiation and skeletal muscle regeneration through the Akt signaling pathway: UBE2C regulates myogenesis through the Akt signaling pathway

**DOI:** 10.3724/abbs.2024062

**Published:** 2024-04-29

**Authors:** Renqiang Yuan, Xiaorong Luo, Ziyun Liang, Shufang Cai, Yunxiang Zhao, Qi Zhu, Enru Li, Xiaohong Liu, Delin Mo, Yaosheng Chen

**Affiliations:** 1 State Key Laboratory of Biocontrol School of Life Sciences Sun Yat-sen University Guangzhou 510275 China; 2 Guangxi Yangxiang Agriculture and Husbandry Co. Ltd. Guigang 537100 China

**Keywords:** UBE2C, muscle, myoblast differentiation, Akt

## Abstract

Ubiquitin-conjugation enzyme E2C (UBE2C) is a crucial component of the ubiquitin-proteasome system that is involved in numerous cancers. In this study, we find that UBE2C expression is significantly increased in mouse embryos, a critical stage during skeletal muscle development. We further investigate the function of UBE2C in myogenesis. Knockdown of *UBE2C* inhibits C2C12 cell differentiation and decreases the expressions of MyoG and MyHC, while overexpression of *UBE2C* promotes C2C12 cell differentiation. Additionally, knockdown of *UBE2C*, specifically in the tibialis anterior muscle (TA), severely impedes muscle regeneration *in vivo*. Mechanistically, we show that *UBE2C* knockdown reduces the level of phosphorylated protein kinase B (p-Akt) and promotes the degradation of Akt. These findings suggest that UBE2C plays a critical role in myoblast differentiation and muscle regeneration and that UBE2C regulates myogenesis through the Akt signaling pathway.

## Introduction

Skeletal muscle is a highly complex and heterogeneous tissue that has a broad range of functions. Among these processes, myogenesis and skeletal muscle regeneration are two processes involved in muscle generation, including myoblast proliferation, differentiation, and fusion [ 1, 2] . These coordinated events depend on highly complex molecular regulatory networks [3]. The myogenic regulatory factors (MRFs), including myogenic differentiation 1 (MyoD), myogenic factor 5 (Myf5), myogenin (MyoG), and myogenic regulator factor 4 (MRF4), play well-defined roles in muscle development and regeneration [ 4, 5] . In addition, numerous key genes that participate in muscle development have been described in recent years [ 6– 9] . Moreover, signaling pathways are the key link in the regulatory network of muscle differentiation [ 10, 11] . The PI3K/Akt/mTOR pathway was confirmed to be a critical regulator of skeletal muscle differentiation and growth [ 12, 13] . Akt contributes to muscle hypertrophy and myofiber growth in adult muscle without activating the proliferation of muscle satellite cells [14] and stimulates myogenesis by promoting the expressions of MyoD [15] and MyoG [16].

Ubiquitination is an important type of posttranslational modification of proteins that requires ubiquitin-activating enzyme (E1), ubiquitin-conjugating enzyme (E2), and ubiquitin-ligase enzymes (E3), and through three-step sequential actions, it transfers the activated ubiquitin from the E2 to the substrate [ 17, 18] . The ubiquitin-conjugating enzyme E2C is a ubiquitin-binding enzyme that accepts ubiquitin from E1 and transfers it to a substrate associated with E3. Previous studies confirmed that UBE2C plays an important role in various malignancies [ 19, 20] and affects cancers through the PI3K/Akt/mTOR signaling pathways [ 21– 23] . However, little is known about the function and molecular mechanisms of UBE2C in skeletal muscle.

In the present study, our findings indicated that knockdown of *UBE2C* impedes myoblast differentiation and muscle regeneration. Specifically, inhibiting UBE2C in C2C12 cells results in reduced level of phosphorylated Akt (p-Akt) and accelerated Akt degradation. These observations provide compelling evidence for the critical involvement of UBE2C in the regulatory mechanisms underlying myogenesis by regulating the Akt signaling pathway.

## Materials and Methods

### Cell culture

C2C12 myoblasts were purchased from the American Type Culture Collection (ATCC, Manassas, USA) and cultured in Dulbecco’s modified Eagle’s medium (DMEM; Corning, New York, USA) supplemented with 10% fetal bovine serum (FBS), 100 U/mL penicillin and 100 U/mL growth medium (GM). To induce differentiation, the cells were switched to DMEM supplemented with 2% horse serum (differentiation medium, DM) after they reached 100% confluence. All cells were cultured in a 37°C incubator with 5% CO _2_.

### Animals

Eight-week-old female C57BL/6 mice were housed under SPF conditions with a 12/12-h dark/light cycle and *ad libitum* access to food and water. All the experimental procedures were approved by the Animal Care and Use Committee of Guangdong Province and were carried out according to ethical standards. The approval ID is SYSU-IACUC-2020-B0614.

### Western blot analysis

Total proteins were extracted from C2C12 cells or mouse tissues using RIPA buffer supplemented with 1 mM PMSF (GenStar, Beijing, China). The collected proteins were subjected to 10% or 12% SDS-PAGE and transferred to 0.22-μm or 0.45-μm PVDF membranes (Millipore, Billerica, USA), which were blocked with 3% bovine serum albumin (BSA) in 0.1% TBS-Tween for 1 h at room temperature and then incubated with primary antibodies overnight at 4°C. After being incubated with appropriate HRP-conjugated secondary antibodies, the blots were visualized with an enhanced chemiluminescence (ECL) detection kit (FDbio, Hangzhou, China). The antibodies used are described in Supplementary Table S1.

### Cardiotoxin (CTX) injury and intramuscular transfection of siRNAs

The intramuscular transfection of siRNAs was performed using an Entranster- *in vivo* kit (Engreen, Beijing, China). The sequences of the siRNAs and the siRNA transfection system used *in vivo* are shown in Supplementary Tables S2 and S3. The hindlimbs of six 8-week-old female mice were cleaned with 75% alcohol. Then, the mixture containing si-UBE2C was injected into the left TA muscles, and the mixture containing NC was injected into the right TA muscles as a negative control.

CTX (Sigma, St Louis, USA) was dissolved in sterile saline to a final concentration of 20 mM. One day after siRNA injection, the hindlimbs of the mice were cleaned with alcohol, and the tibialis anterior (TA) muscles were intramuscularly injected with 50 μL of CTX via a hypodermic syringe. To maintain the long-term effects of siRNA, si-UBE2C or NC was injected into the TA muscle every 2 days. Regenerating TA muscles were isolated at 3, 7, and 14 days after CTX injection.

### RNA extraction and real-time quantitative PCR

Total RNA was extracted from C2C12 cells and mouse tissues with Trizol reagent (Invitrogen Carlsbad, USA) and reverse transcribed to cDNA using StarScript II First-strand cDNA Synthesis Mix (GenStar). Real-time quantitative PCR was performed using 2× RealStar Green Power Mixture (GenStar) on a QuantStudio 7 Flex (ABI, Foster City, USA). *GAPDH* was used as an internal control for normalization. The primers used for qPCR are listed in Supplementary Table S4.

### Transfection of plasmids and siRNA

Three siRNAs targeting UBE2C (si-UBE2C-1, si-UBE2C-2, and si-UBE2C-3) were purchased from Invitrogen. The sequences of all siRNAs are listed in Supplementary Table S5. For the UBE2C overexpression vector, the coding sequences (CDSs) of the mouse *UBE2C* gene were inserted into the pcDNA3.1 vector (Invitrogen). C2C12 cells were seeded into 12-well plates 24 h before treatment and then transfected with siRNAs using Lipofectamine 3000 (Invitrogen) or with overexpression plasmids using Y40 (Invitrogen).

### Immunofluorescence (IF) staining

C2C12 cells were fixed with 4% paraformaldehyde for 10 min, permeabilized in 0.5% Triton X-100 for 15 min, blocked in 3% BSA/PBST for 1 h, incubated with indicated primary antibodies and the corresponding fluorescein-linked secondary antibody listed in Supplementary Table S1, and then counterstained with 4′,6-diamidino-2-phenylindole (DAPI; 1:1000 in PBS). Images were captured with a fluorescence reverse microscope (Nikon, Tokyo, Japan). The differentiation rate was calculated as the percentage of nuclei in MyHC-positive cells. Nine images of immunofluorescence from three replicates (three images from each replicate) were randomly selected for analysis in each group.

### Use of SC79 and LY294002

C2C12 cells were treated with siRNA or overexpressed plasmid, and cultured on growth medium. The 10 μM SC79 (Selleck, Housto, USA) and 20 μM LY294002 (Selleck) were added to the interfering or overexpressed cells 12 h later, respectively. After cells reached 100% confluence the cells were switched to differentiation medium to induce differentiation.

### 5-Ethynyl-2′-deoxyuridine (EDU) assay

The EdU assay was performed using an EDU kit (RiboBio, Guangzhou, China). C2C12 cells cultured in GM for 36 h were treated with 50 mM EdU for 2 h. Then, the C2C12 cells were fixed, permeabilized, and stained with Apollo 567 (RiboBio) according to the manufacturer’s protocol. The nuclei were stained with DAPI. Images were captured with a fluorescence reverse microscope (Nikon).

### Hematoxylin and eosin (H&E) staining

TA muscles were fixed in 4% formalin for 24 h, dehydrated with graded ethanol, embedded in paraffin, and sectioned at 4 μm. Muscle sections were dewaxed using xylene and then rehydrated with graded ethanol and double distilled water. TA muscle paraffin sections were stained using an H&E staining kit (Xiuwei, Guangzhou, China) according to the manufacturer’s instructions. Images were captured with a confocal microscope (Leica, Wetzlar, Germany).

### Statistical analysis

Data are presented as the mean±SEM, and the significance of differences was analyzed using the unpaired two-tailed Student’s *t* test. *P*<0.05 was considered to indicate statistical significance.

## Results

### The expression of UBE2C is downregulated during myogenesis

**Figure 1:**
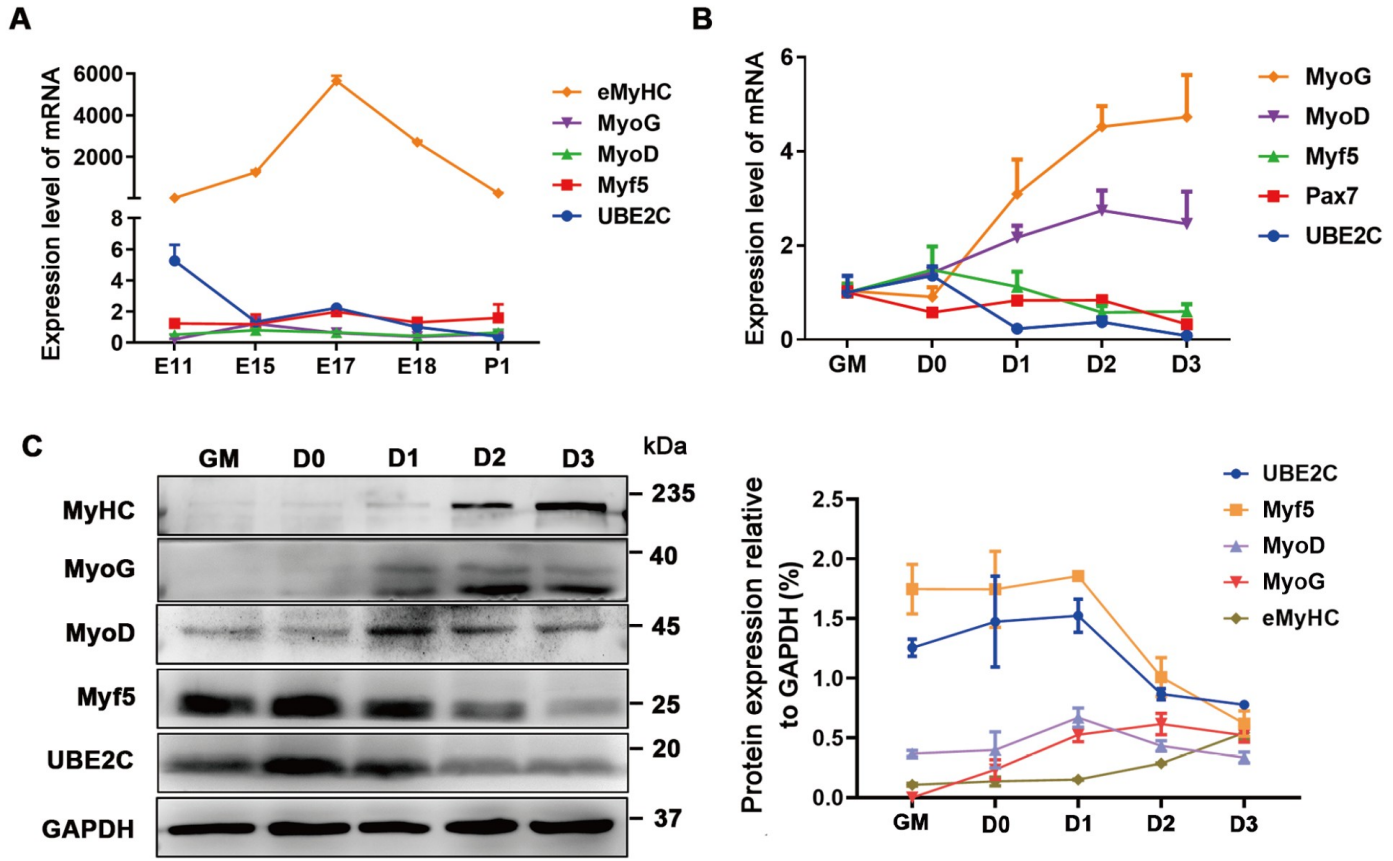
Figure 1 Expression pattern of UBE2C (A) The mRNA expressions of UBE2C and myogenic markers were measured in the longissimus dorsi of mice at five developmental stages. GAPDH was used as an internal control for normalization. E denotes the embryonic period, while P indicates post-birth. (B) The mRNA levels of UBE2C and myogenic markers were evaluated at five time points during C2C12 differentiation. (C) The protein expression levels of UBE2C and myogenic markers were evaluated at five time points during C2C12 differentiation (left). Grayscale scanning for quantifying protein expression (right). The cells were cultured in growth medium (GM) at sub-confluent densities. When the cells reached 100% confluence, it was defined as day 0 (D0), and the growth medium was changed to differentiation medium. Data are presented as the mean±SEM ( n=3 per group).

To determine the role of UBE2C in myogenesis, the expression profiles of muscle samples from the longissimus dorsi at five developmental stages were analyzed. qPCR analysis revealed that the expression level of *UBE2C* was greater during the embryonic stage than that in the postnatal stage and displayed a trend similar to that of *Myf5* ( Figure 1A). Additionally, both the mRNA and protein levels of UBE2C were gradually decreased from proliferation to differentiation in C2C12 cells ( Figure 1B,C). These observations collectively suggested that the expression pattern of UBE2C may be involved in myogenesis.

### UBE2C is essential for C2C12 differentiation

Given that UBE2C is highly expressed in embryonic longissimus dorsi and proliferating cells ( Figure 1), UBE2C may affect the proliferation of myoblasts. However, the real-time cell proliferation assay, EdU labelling, and Ki67 immunofluorescence staining all indicated no significant difference in proliferation between C2C12 cells treated with siRNA and control cells ( Supplementary Figure S1C–E). Notably, we observed an obvious decrease in the expression of Myf5, the myogenic regulatory factor expressed at the earliest stage [24], after *UBE2C* knockdown ( Supplementary Figure S1A,B), suggesting a potential role for UBE2C in myogenesis.

**Figure 2:**
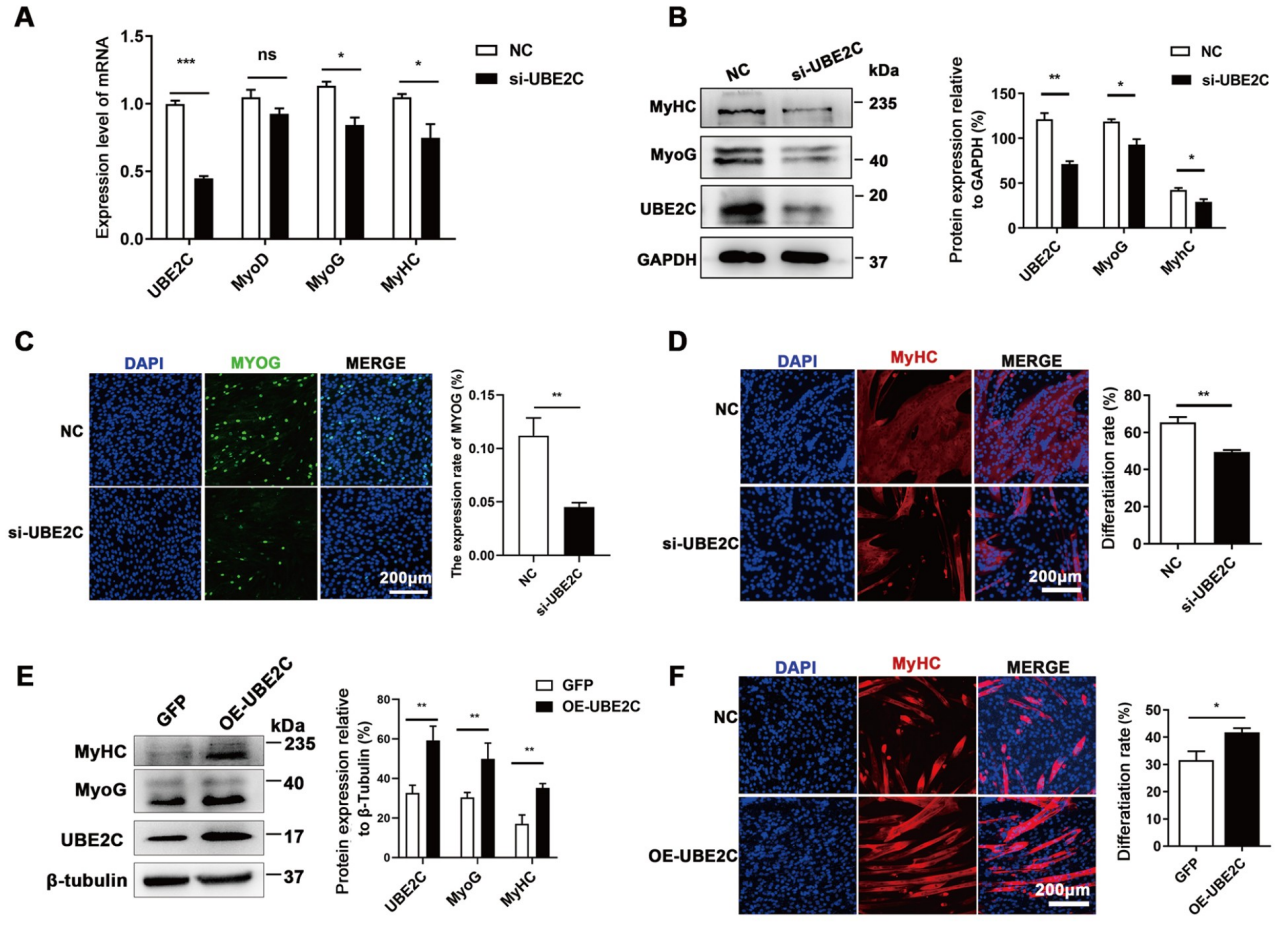
Figure 2 UBE2C is essential for C2C12 differentiation (A) The mRNA expression levels of UBE2C, MyoD, MyoG, and MyHC were quantified using qPCR one day after the induction of differentiation. (B) Western blot analysis was used to determine the protein expression levels of UBE2C, MyoG, and MyHC (left panel). Greyscale scanning of the western blots is shown in the right panel, with GAPDH serving as the loading control. (C) Immunofluorescence staining for MyoG was performed on C2C12 cells one day after induction of differentiation (left panel), and the percentage of MyoG-positive cells was calculated (right panel). (E) Western blot analysis was utilized to assess the protein expression levels of UBE2C, MyoG, and MyHC in cells overexpressing UBE2C (left panel). Greyscale scanning of the western blots is shown in the right panel. Immunofluorescence staining for MyHC was conducted three days after induction of differentiation in cells with UBE2C knockdown (D) or overexpression (F). The nuclei were counterstained with DAPI. Scale bar: 200 μm. Data are presented as the mean±SEM, n=3 per group. * P<0.05, ** P<0.01, *** P<0.001, and ns indicates no significant difference.

Meanwhile, decreased mRNA and protein levels of MyoG and MyHC were observed ( Figure 2A,B). Immunofluorescence staining for myoG and MyHC revealed a reduction in the number of myoG ^+^cells and myotubes, respectively ( Figure 2C,D). Conversely, when UBE2C was overexpressed in C2C12 cells, the expression levels of MyoG and MyHC increased ( Figure 2E). Additionally, UBE2C overexpression led to enhanced C2C12 cell differentiation, as evidenced by a significant increase in the number of multinucleated myotubes ( Figure 2F). Collectively, these results indicated that UBE2C promotes myoblast differentiation.

### UBE2C promotes myoblast differentiation by regulating Akt phosphorylation and degradation

**Figure 3:**
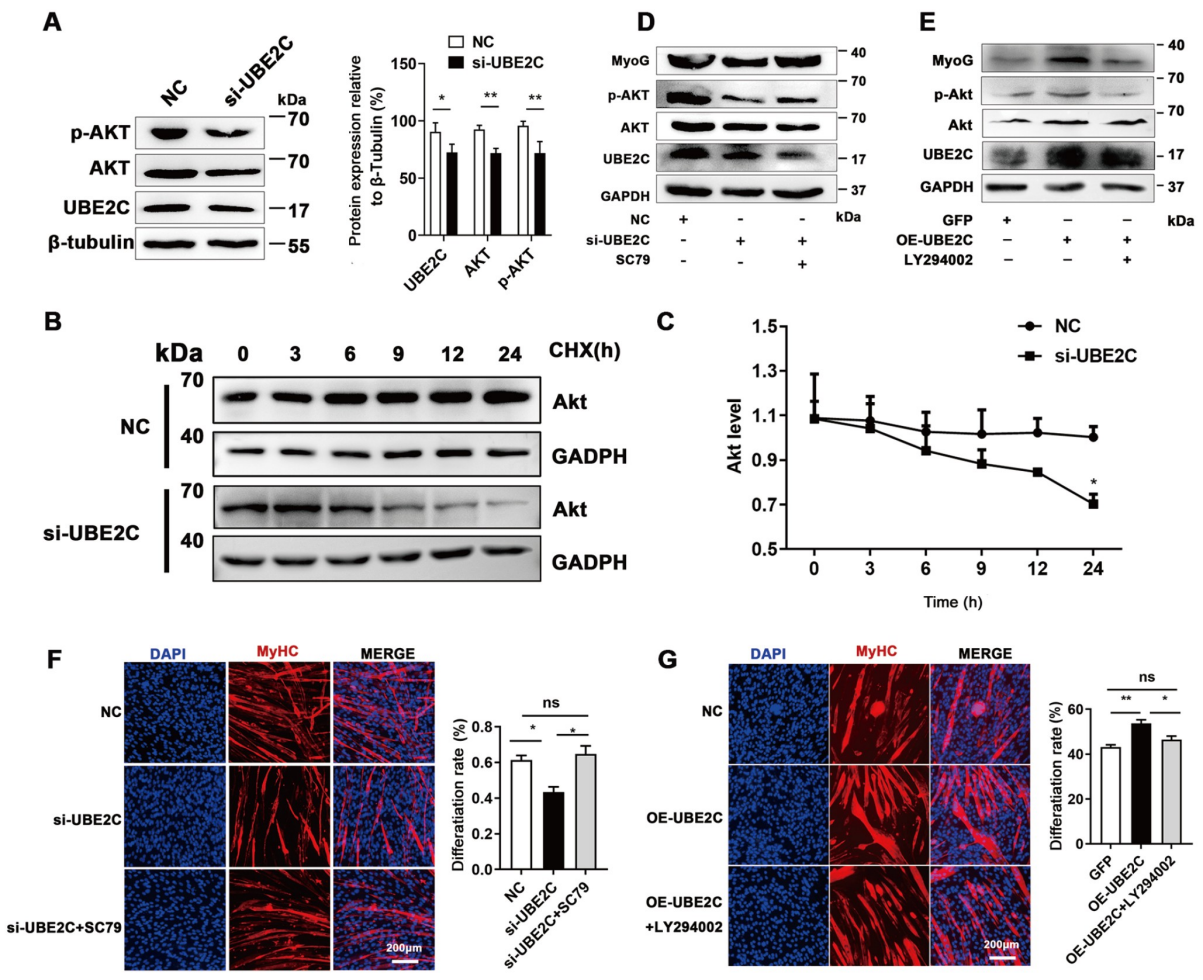
Figure 3 UBE2C regulates Akt phosphorylation and degradation (A) The protein levels of Akt and p-Akt were analyzed by western blot analysis. (B) C2C12 cells were transfected with NC or si-UBE2C, followed by incubation with CHX. The cells were harvested at the designated time points for western blot analysis to evaluate Akt protein levels. (C) Greyscale scanning of the western blots was used to quantify the Akt protein levels. (D) C2C12 cells were transfected with siRNA and treated with SC79. The expression levels of MyoG, MyHC, Akt, and p-Akt were detected by western blot analysis. (E) C2C12 cells were transfected with plasmids to overexpress UBE2C and treated with LY294002. The expression levels of MyoG, MyHC, Akt, and p-Akt were analyzed by western blot analysis. (F,G) Immunofluorescence staining was performed on C2C12 cells treated as described in (D,E), with detection specifically targeting MyHC. Scale bar: 200 μm. Data are presented as the mean±SEM, n=3 per group. * P<0.05, and ** P<0.01. ns indicates no significant difference.

The PI3K/Akt signaling pathway is widely recognized as one of the pivotal pathways involved in myogenesis [ 25, 26] . In this study, inhibiting UBE2C expression led to a decrease in Akt expression and phosphorylation ( Figure 3A). Furthermore, we evaluated the protein stability of Akt using cycloheximide (CHX) treatment. The results demonstrated that UBE2C inhibition promoted the degradation of the Akt protein in C2C12 cells ( Figure 3B,C), suggesting that *UBE2C* knockdown reduced Akt protein stability.

To further investigate whether UBE2C regulates myoblast differentiation through the modulation of Akt phosphorylation, C2C12 cells were treated with SC79, an agonist of the PI3K/Akt signaling pathway, and LY294002, a PI3K signaling inhibitor. The results showed that SC79 restored MyoG expression and rescued the defects in myoblast differentiation caused by *UBE2C* knockdown ( Figure 3D,F). Conversely, the promoting effects of UBE2C overexpression on MyoG expression and myoblast differentiation were abolished by LY294002 treatment ( Figure 3E,G). These findings collectively indicate that Akt phosphorylation facilitates myoblast differentiation and suggest that *UBE2C* knockdown inhibits myogenic differentiation by impairing Akt phosphorylation and enhancing Akt degradation.

### *UBE2C* knockdown blocks muscle regeneration

**Figure 4:**
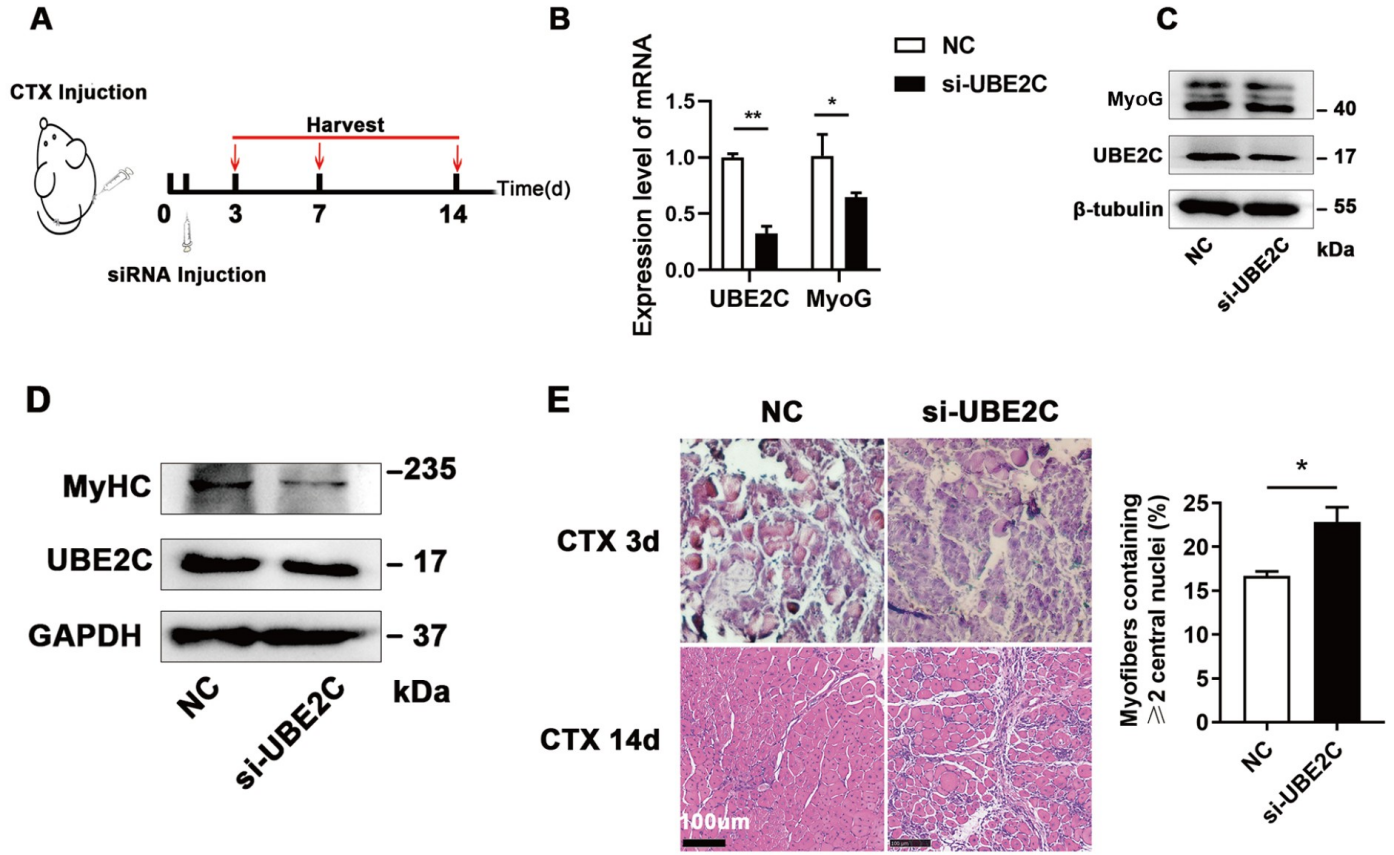
Figure 4 *UBE2C* knockdown restrains skeletal muscle regeneration (A) The mouse model of TA tissue injury is illustrated in the schematic diagram. (B,C) After 7 days, the mRNA (B) and protein (C) expression levels of the given genes were evaluated in mice subjected to TA treatment with CTX. (D) The protein expression level of MyHC was assessed after 14 days of CTX treatment. (E) H&E staining was conducted on TA samples harvested at 3 d and 14 d after CTX treatment (left panel), and the proportion of muscle fibers with two or more central nuclei were quantified (right panel). Scale bar: 100 μm. Data are presented as the mean±SEM, n=3 per group. * P<0.05, and ** P<0.01.

To investigate whether the functions of UBE2C in C2C12 cells can be repeated *in vivo*, we utilized a CTX-mediated muscle regeneration model. Si-UBE2C or negative control (NC) was injected into TA muscles every 2 days to maintain *UBE2C* knockdown efficiency. Subsequently, the TA muscles were harvested on days 3, 7, and 14 ( Figure 4A). The expression of MyoG was significantly lower in the TA muscles of the si-UBE2C-treated group than in those of the NC group on day 7 ( Figure 4B,C), which is consistent with the *in vitro* experimental results ( Figure 2B). Additionally, *UBE2C* knockdown resulted in a reduction in MyHC protein level on day 14 ( Figure 4D). H&E staining revealed impeded formation of myofibers on day 14 in the UBE2C inhibition group ( Figure 4E). These findings align with the *in vitro* observations, indicating that *UBE2C* knockdown inhibits MyoG expression and myoblast differentiation, consequently leading to delayed muscle tissue repair and regeneration.

## Discussion

Skeletal muscle originates from the embryonic paraxial mesoderm [ 2, 4] . Myofiber formation in the embryonic stage is crucial for the growth of limbs and trunks [27] and the muscle mass of livestock and poultry [28]. In this study, we observed that the expression of UBE2C in embryonic skeletal muscle was greater than that in postnatal skeletal muscle in mice. Furthermore, UBE2C was found to be expressed at a higher level during proliferation rather than terminal differentiation. These findings suggest a potential role for UBE2C in myoblast proliferation. However, real-time cell proliferation assays, EdU labelling and Ki67 immunofluorescence staining all showed that knockdown of *UBE2C* did not affect proliferation, although there was a significant decrease in *Myf5* expression. This prompted us to explore the function of UBE2C in differentiation.

Our results verified that UBE2C is essential for myoblast differentiation. *UBE2C* knockdown decreased the expression of MyoG and suppressed myoblast differentiation in C2C12 cells, while UBE2C overexpression had the opposite effect. Consistently, intramuscular injection of si-UBE2C suppressed MyoG expression and blocked myoblast differentiation during CTX-mediated muscle regeneration.

The PI3K/Akt/mTOR signaling pathway is a well-established pathway involved in various cellular processes, including apoptosis, proliferation, differentiation, and metabolism [29]. In this study, we observed that knockdown of *UBE2C* resulted in a decrease in the expression levels of both total Akt and phosphorylated Akt. After UBE2C was overexpressed in C2C12 cells, the total and phosphorylated Akt levels increased. Previous studies have extensively reported the involvement of UBE2C in cancer through the Akt/mTOR signalling pathway. These studies consistently demonstrated that *UBE2C* knockdown is accompanied by inhibition of p-Akt [ 21– 23] . The precise activation and degradation of Akt play important roles in maintaining diverse biological responses [ 30, 31] . Two distinct ubiquitination systems have been reported to regulate Akt signalling [32]. Wei *et al*. [33] reported that dephosphorylation of p-Akt could accelerate its degradation through the ubiquitin-proteasome pathway. In our study, inhibition of UBE2C facilitated the protein degradation of Akt. Based on these findings, we propose that UBE2C may regulate myogenesis by modulating the stability and phosphorylation of Akt.

**Figure 5:**
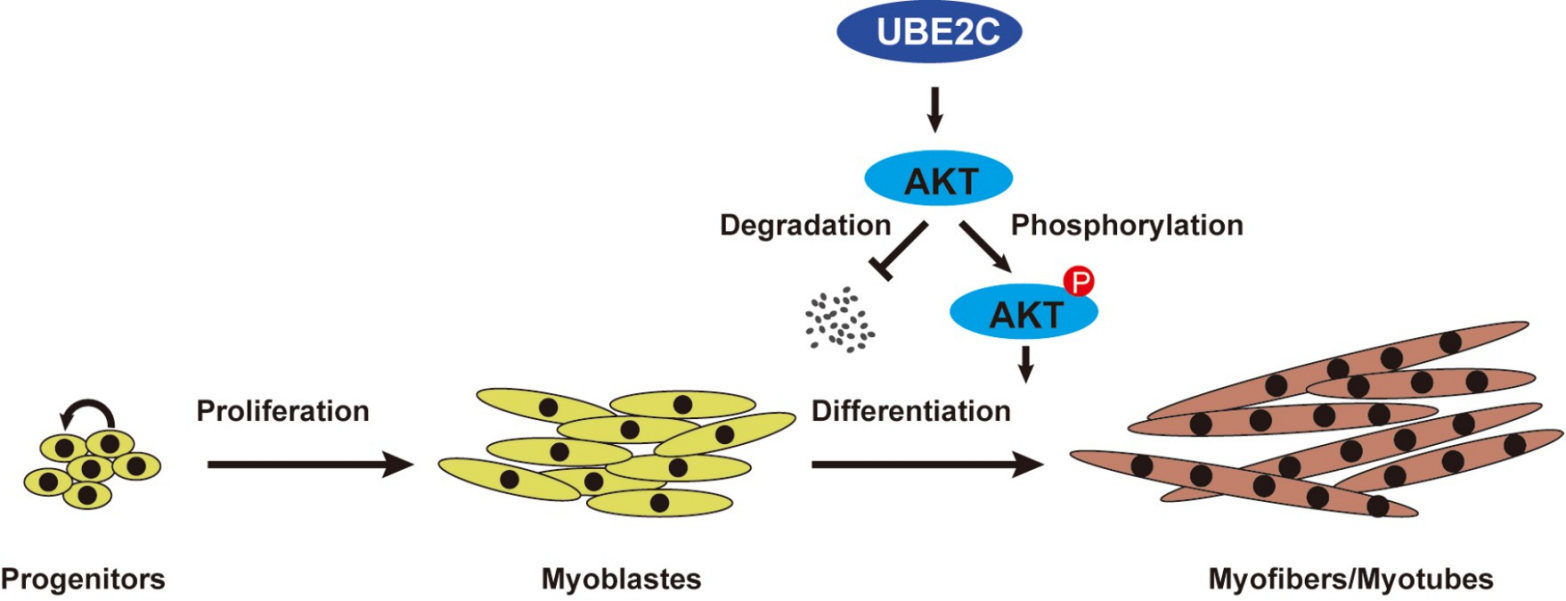
Figure 5 Schematic diagram of UBE2C regulating myogenesis UBE2C promotes myoblast differentiation and muscle regeneration by regulating Akt phosphorylation and stability.

In conclusion, our study provides novel evidence supporting the crucial role of UBE2C in myoblast differentiation and skeletal muscle regeneration ( Figure 5). Specifically, we elucidated that UBE2C enhances Akt phosphorylation and stabilizes Akt, thereby promoting C2C12 cell differentiation.

## Supporting information

23538Supplementary_material

## Supplementary Data

Supplementary data is available at *Acta Biochimica et Biophysica Sinica* online.
