# Supplementary material for: UBE2C promotes myoblast differentiation and skeletal muscle regeneration through the Akt signaling pathway: UBE2C regulates myogenesis through the Akt signaling pathway
Source: Acta Biochim Biophys Sin (Shanghai). 2024 Apr 29;56(7):1065–71. doi: 10.3724/abbs.2024062 (PMC11322864; doi:10.3724/abbs.2024062)
Supplement: 23538Supplementary_material [file 23538Supplementary_material.pdf]

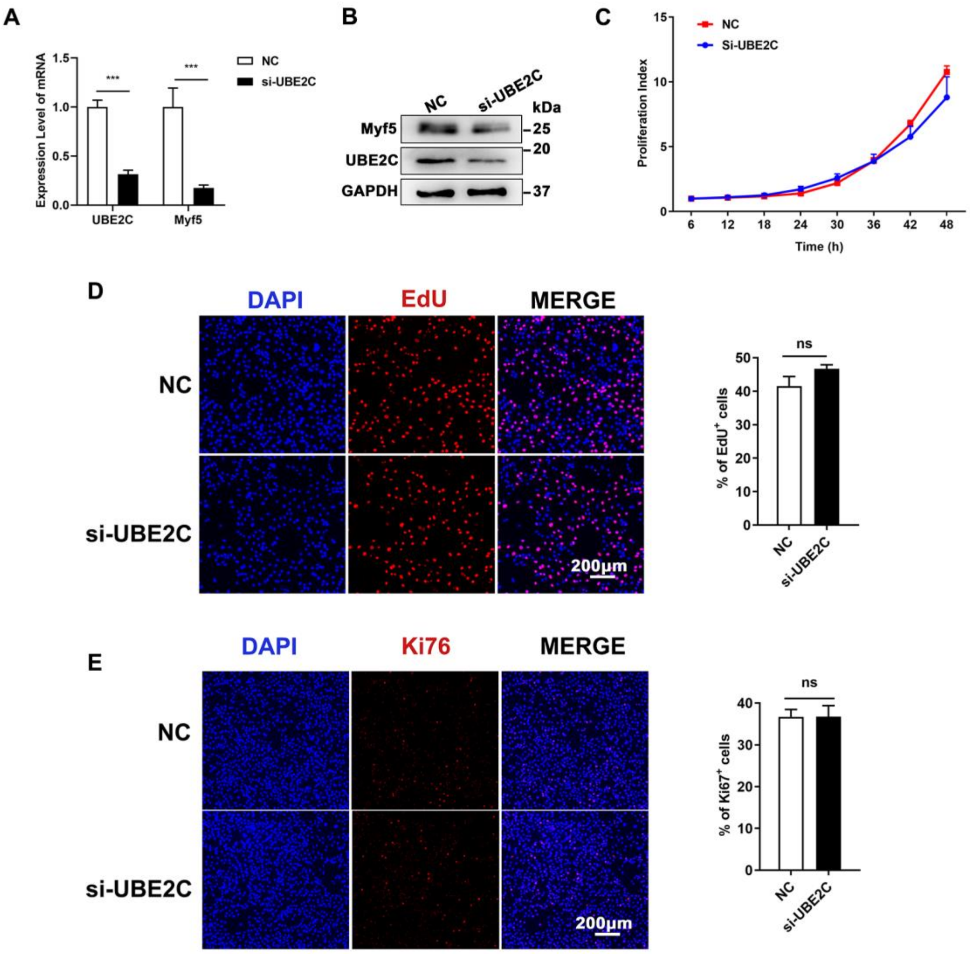

Supplementary Figure S1. UBE2C did not influence C2C12 proliferation (A) The mRNA expression level of *Myf5* was assessed by qPCR in the proliferation phase of cells treated with si-UBE2C. (B) Western blot analysis was performed to determine the protein level of Myf5 in proliferating C2C12 cells treated with si-UBE2C. (C) C2C12 cells were transfected with si-UBE2C for 24 h, followed by real-time proliferation assay conducted at regular intervals of 6 h for a total duration of 48 h. Data points were recorded and analyzed. (D) After 24 h of si-UBE2C transfection, C2C12 cells were subjected to EdU staining to assess cell proliferation. The percentage of EdU-positive cells relative to the total number of nuclei was quantitated and presented in a statistical graph. (E) Following 24 h of si-UBE2C transfection, immunofluorescence staining for Ki67 was performed in C2C12 cells. The percentage of Ki67-positive cells relative to the total number of nuclei was quantified and presented in a statistical graph. The nuclei were counterstained with DAPI. Scale bar: 200 μm. Data are presented as the mean ± SEM. *P<0.05, **P<0.01, ***P<0.001. ns indicates no significant difference.

Supplementary Table S1. The antibodies used in this study

Antibody	Company	Catalogue	WB	IF
Anti-UBE2C antibody	CST	14234	1/1000	
Anti-Myf5 antibody	Abcam	125301	1/1000	
Anti-MyoG antibody	Abcam	124800		1/500
Anti-MyoG antibody	Abcam	1835	1/1000	
Anti-MyHC antibody	Abcam	51263	1/1000	1/500
Anti-Akt antibody	CST	9242	1/1000	
Anti-pAkt antibody	CST	4060	1/1000	
Anti-β-tubulin antibody	CST	2146	1/1000	
Anti-GAPDH antibody	Abcam	9485	1/1000	
Anti-Ki67 Antibody	Abcam	15580	1/1000	
Anti-rabbit IgG HRP-linked antibody	CST	7074	1/2000	
Anti-mouse IgG HRP-linked antibody	CST	7076	1/2000	
Anti-rabbit IgG (H+L), (Alexa Fluor® 488Conjugate)	CST	4412		1/1000
Anti-mouse IgG (H+L), (Alexa Fluor® 555Conjugate)	CST	4409		1/1000

Supplementary Table S2. The siRNA sequence of UBE2C used in *vivo*

siRNA	Sequence (5′→3′)
si-UBE2C	CUCAUGACAUCUGGUGACATT<br>UGUCACCAGAUGUCAUGAGTT

Supplementary Table S3. Transfection system of siRNA in *vivo*

	Composition	Volume
A buffer	sterile saline	12.5 μL
	siRNA/NC (1μg/μL)	12.5 μL
B buffer	sterile saline	18.75 μL
	Entranster-in *vivo*	6.25 μL

Supplementary Table S4. Sequences of primers used for qPCR in this study

Gene	Primer sequence (5′→3′)
UBE2C	Forward: GAGTCAGACAACCTGTTCAAGTG<br>Reverse: TCTAGGGAGAGTTTGTACCTCAG
GAPDH	Forward: AGGTCGGTGTGAACGGATTTG<br>Reverse: TGTAGACCATGTAGTTGAGGTCA
MyoD	Forward: CCACTCCGGGACATAGACTTG<br>Reverse: AAAAGCGCAGGTCTGGTGAG
MyoG	Forward: GAGACATCCCCCTATTTCTACCA<br>Reverse: GCTCAGTCCGCTCATAGCC
Pax7	Forward: TCTCCAAGATTCTGTGCCGAT<br>Reverse: CGGGGTTCTCTCTCTTATACTCC
Myf5	Forward: AAGGCTCCTGTATCCCCTCAC<br>Reverse: TGACCTTCTTCAGGCGTCTAC
Akt	Forward: ATGAACGACGTAGCCATTGTG<br>Reverse: TTGTAGCCAATAAAGGTGCCAT

Supplementary Table S5. The siRNA sequences of UBE2C used in *vitro*

siRNA	Sequence (5′→3′)
si-UBE2C-1	GCAAGAAACCUAUUCAAAGTT<br>CUUUGAAUAGGUUUCUUGCTT
si-UBE2C-2	CUCAUGACAUCUGGUGACATT<br>UGUCACCAGAUGUCAUGAGTT
si-UBE2C-3	GAGUCAGACAACCUGUUCATT<br>UGAACAGGUUGUCUGACUCTT
NC	UUCUCCGAACGUGUCACGUTT<br>ACGUGACACGUUCGGAGAATT
